# Psychiatric Nurses’ Perceptions about Physical Restraint; A Qualitative Study

**Published:** 2014-01

**Authors:** Malek Fereidooni Moghadam, Masoud Fallahi Khoshknab, Mehrnoosh Pazargadi

**Affiliations:** 1 Department of Psychiatric Nursing, Ahvaz Jundishapur University of Medical Sciences, Ahvaz, Iran;; 2 Department of Nursing, University of Social Welfare and Rehabilitation Sciences (USWR), Tehran, Iran;; 3 Department of Nursing Management, Shahid Beheshti University of Medical Sciences, Tehran, Iran

**Keywords:** Physical Restraint, Mental Illness, Nurses, Psychiatric Ward, Iran

## Abstract

**Background:** The use of physical restraint as an intervention in the care of psychiatric patients dates back to the beginning of psychiatry. Although it is a challenging question, it is still one of the common procedures in psychiatry. Considering that very little research has been done in Iran in relation to physical restraint, this qualitative study aimed to investigate the experiences of  nurses working in psychiatric wards regarding physical restraint.

**Methods: **This qualitative study was done on 14 nurses working in the psychiatric hospitals of Ahvaz city, southern Iran, during 2011-2012. The participants were selected by purposive sampling. Semi-structured interviews were used for data collection, which were continued until data saturation and emergence of themes. Inductive content analysis was used to analyze the data.

**Results: **Four categories emerged: (1) Restraint as a multi-purpose procedure, (2) Processing of physical restraint, (3) Restraint as a challenging subject and (4) The effects of restraint on the spectrum. Each category has several different sub-categories.

**Conclusion: **The participants described using physical restraint as one of the main strategies to control psychiatric patients, and despite having negative consequences, it is extensively used. Given the risks and challenges of using physical restraint, nursing education should find alternative methods.

## Introduction

The use of physical restraint as an intervention in the care of psychiatric patients dates back to the beginning of  the science of psychiatry.^1^ However, it is still one of the challenging questions in the psychiatric services^2^ and has always been considered as a moral argument.^1^

Physical restraint includes devices designed to limit a patient’s physical movements such as limb holders, safety vests and bandages. It is used to handle violent and maladaptive behaviors,^3^ manage patients with severe mental disorders,^4^ prevent injury and reduce agitation and aggression.^3^

This controlling intervention, which has been inherently designed to protect patients from harm to themselves or others, is associated with many potential complications. Studies have shown the negative effects of physical restraint on both patients and personnel.^5^ Some of these effects in patients include risks of physical injury and death,^6^ negative emotional impact on the patients and their family members,^7^ experiencing psychological distress,^5^ further stimulation of aggression and damage to therapeutic alliances between the patients and the staff.^8^ On the other hand personnel are at risk of physical injury, emotional effects,  and  death.^7^ Although this intervention  can be effective as a last resort in preventing injury to patients and maintaining their security, it is considered to be traumatizing to patients and contrary to treatment principles and patient dignity.^6^ Thus the use of coercive interventions is one of the indicators of the quality of psychiatric treatment in hospitalized patients, and many attempts have been conducted in different countries for early  prevention together with alternative  interventions to reduce and eliminate the use of these approaches.^6^

Despite extensive literature on the potential complications of using physical restraint, it is still considered as a permanent and effective intervention in the management of unpleasant behaviors on acute and long-term care environments.^9^ Studies show that in many countries more than 20% of psychiatric patients are restrained physically in a period during their hospitalization.^6^

Researchers who have tried to understand and explain the use of physical restraint in psychiatric institutions, have identified several predictor variables in the use of restraint. These variables include: poor staff and facility standards, inadequate training of staff, poor education level of staff, inadequate treatment programs, staff’s attitude about physical restraint, and hospital management.^10^ Moreover, clinical factors such as demographic characteristics or diagnosis of patients are also influential.^11^ In general, empirical studies have shown that the rate, duration and application of seclusion and physical restraint are different in various psychiatric hospitals  even under the same operational policies and guidelines.^12^

Numerous qualitative studies have been conducted on the use of physical restraint. In an ethnographic study, Marangos-Frost and Wells studied the psychiatric nurses’ thoughts and feelings about the use of physical restraint. The results identified four themes: (1) framing of the situation (potential for imminent harm), (2) unsuccessful search for alternatives to physical restraint, (3) conflicted nurse, and (4) contextual conditions of restraint. The results also indicated that the use of restraint is more complex than is currently conveyed in the literature. They suggested that further investigation was necessary to draw definitive conclusions about the continued use of physical restraint in the care of patients in psychiatric units.^13^

Very few studies have been conducted in Iran about the use of physical restraint in psychiatric wards. However, according to the researchers’ experiences, the use of physical restraint in the psychiatric wards of Iran to control violent and non-adaptive behaviors of patients is common. In a study in Iran, the researchers compared the nurses’ physical restraint methods with the existing standards in psychiatric wards. While emphasizing on the popularity of physical restraint for controlling the patients’ violent behaviors, they reported that the principles of physical restraint of patients in Iran are far from the related standards.^14^

Given the conflicting results of quantitative studies about the related factors of restraint^7^ and the importance of conducting qualitative studies for the better understanding of a phenomenon,^15^ and also according to another study, the use of physical restraint is still common and should be the focus of further research.^16^ Since the documentation focusing on seclusion  and restraint of patients can be important to provide information for planning alternative models^6^ and that very little research has been conducted in relation to physical restraint in Iran, we aimed to investigate the psychiatric nurses’ experiences of using physical restraint in the psychiatric wards of Ahvaz hospitals, southern Iran.

## Materials and Methods

This qualitative content-analysis study was conducted during 2011-2012. The inclusion criteria for selecting the participants were: 1- Have a bachelor degree or higher in nursing, 2- Having at least 6 months of work experience in psychiatric wards, and 3- Willingness to participate in the study. Ultimately 14 nurses of four psychiatric hospitals, with a bachelor’s degree or higher in nursing, were selected using the purposive sampling method. 

*Data Collection and Analysis*

Audio-recorded, face-to-face, semi-structured interviews were used for data collection. The key question was: “What is your experience of physical restraint in this ward?”  All of the recorded interviews were listened to carefully during the 24 hours after recording and transcribed. The duration of interviews was between 30 to 50 minutes. The interviews were conducted by the first author in Persian language, which were then translated into English. The data collection and analysis preceded concurrently using inductive the content analysis approach. For this purpose, the interviews were transcribed verbatim. After reading the interviews several times, they were divided into meaning units, and after condensation, the condensed meaning units were abstracted and labeled with codes. The codes, based on similarities and differences, were classified into sub-categories and categories. Finally, based on the underlying main idea of the interviews, main categories or themes were extracted. Data collection continued until data saturation and emerging of themes.^17^

To verify the validity (credibility) of the obtained data, the following measures were adopted: 1- prolonged engagement and meetings with the participants, 2- repeated reading of the interviews and drowning in the data, 3- using comments and suggestions of the colleagues to verify categories and 4- finally, returning the coded interviews to some of the participants to attain the consensus among the researchers and the participants in the codes.

*Ethical Considerations*

The Ethics Committee of Ahvaz Jundishapur University of Medical Sciences approved the study. Formal authorization was obtained from the College of Nursing and Midwifery of Ahvaz Jundishapur University of Medical Sciences and the hospitals for both the sampling and the study. Both the purpose and method of the research were described for the participants, and written informed consent to participate in the study was received from all of them.

## Results

The participants in the study were 14 nurses with a mean±SD age of 40.42±8.58 (range: 25-52 years), who had a work experience in nursing of 7 months to 29 years, and experience in the psychiatric wards from 7 months to 26 years.  

The data analysis revealed four main categories: (1) Restraint as a multi-purpose procedure, (2) Processing of physical restraint, (3) Restraint as challenging subject and (4) The effects of restraint on the spectrum. Each category or theme has different sub-categories and minor classes (table 1).

**Table 1:** Themes, categories and sub-categories of psychiatric nurses experiences of physical restrain in psychiatric ward

**Themes**	**Categories**	**Sub-categories**
Restraint as a multi-purpose procedure	Controlling restraint	
	Treatment auxiliary restraint	
	Preventive restraint	
Processing of physical restraint	Before the restraint	Preparation
		Assessment
	During the restraint	Surrounding the patient
		Reducing the resistance
		Safety restraint
		Non-standard control
		Violent restraint
	After the restraint	Liberating interaction
		Patient enlightenment
		Assessment of the restrained patient
Restraint as challenging subject	Opening the restraint	
	Resistance to restraint	
The restraint effects on the spectrum	Negative effects	Damage to the relationship
		Damage to the patient
		Affected nurse
	Positive effects of caring

1. *Restraint as a Multi-Purpose Procedure*
  The nurses used physical restraint as an acceptable tool and intervention for different purposes in the ward.  These purposes can be placed in the following sub-classes:
  - Controlling restraint
    One of the main applications of physical restraint, according to the participants, is the control of patients such that it is considered as a very important tool for the ward management:
    “Well, our first control in this ward is fixation. (P. 10).
    “Our main weapons in this ward for controlling of the patients are sedative injection and fixation” (P. 6).
  - Treatment auxiliary restraint
    Here, physical restraint is used to speed up access to treatment goals and help the pharmaceutical interventions. One of the participants says:
    “We may fix a patient for a quarter of an hour; it depends on the patient, for example, our patients receive drugs or we inject a tranquilizer. Then they become sleepy and should go to sleep; at this time we fix them.  Maybe after 10 minutes, the patient goes to sleep quickly, then we remove the fixation” (P. 12).
    Also, another application of physical restraint is as a therapeutic measure in order to deal with some patients’ symptoms:
    “As soon as the patient washes his/her hands, and wants again to looking for his/her obsessive works, we have to fix them, if the drugs do not work” (P. 6).
  - Preventive restraint
    It prevents damage to the patients due to disorientation, dizziness and sleepiness, which are mainly caused by the consumption of psychotropic drugs:
     “Sometimes the patient is affected as a result of drug complications; he/she becomes slack and does not know what to do and where to go. Then we fix him/her, because we do not want to cause damage to his/her head or hurt him/her” (Part. 3).
2. *Processing of Physical Restraint*
  This is another main extracted theme in this study. While using physical restraint, the nurses use a set of measures and approaches that, given the processing nature of these approaches, can be placed in three sub-categories:
  - Before restraint
    The nurses, prior to implementing restraint procedures, consider two actions: assessing the patients’ situation for the procedure and also the patient’s needs to be controlled:
     “Upon seeing the patient, by experience, we know whether he/she needs to be fixed or not.” (P. 13).
    “It depends; if [the patient] has antisocial background, he/she is fixed quickly, but if we understand that his/her behavior is not aggressive, he/she is not antisocial, and has no personality disorder, then we do not fix him/her” (P. 3).
    It is worth noting that, according to the participants, using physical restraint requires the physician’s order and the nurse will follow his/her order to control the patient physically:
    “Of course, fixation of the patient to his/her bed has conditions; the doctor’s order comes first” (P. 10).
    After general assessment of the patient, the nurses prepare him/her for the procedure of physical restraint. This preparation mainly includes injection of tranquilizers to sedate the patient, and reducing his/her resistance:
    “Regarding the patient who has to be fixed, we first inject a sedative drug to loose him/her such that he/she cannot resist much; this means that we do not directly fix the patient” (P. 3).
  - During the restraint
    According to the participants, one of the most important stages of physical restraint process is carrying out some practices and approaches as follows:
    Surrounding the patient: this is one of the approaches used by the nurses in physical restraint, which is actually one of the requirements of this procedure:
    “For fixing the patient, you cannot do it yourself alone, even if the patient is too weak or has a small body. I think, at least three or four people are needed to control the patient in order to surround the patient” (P. 11).
    Reducing the patient’s resistance: is of other essential approaches used during the patient restraint:
    “You have to surround the patient; we usually do this and try to handle him/her completely. You should be coordinated with the personnel. One or two people try to take the patient’s hands an hold his/her feet so that the patient is restrained in a very short time without being able to react” (P. 11).
    Safety restraint: is of the important approaches in physical restraint of a patient and, according to them, is a requirement for physical restraint:
    “We have special sponge-like ropes for fixation of the patient. The ropes are laminated to prevent injury when trying to knot the patient’s hands. The patient is fixed and all of his/her four limbs are tied to his/her bed, because if only one hand and/or one leg is tied, the patient may, cause damage to him/herself since she/he is hanged from the bed. So we have to close all limbs of the patient” (P. 12).
    “You must be sure not to cause damage to the patient whom you have restrained” (P. 2).
    Non-standard control is another unpleasant and even unethical approach for physical restraining of patients, done by people who are not nurses (including the workers, the security guards, and even the patients) to restraint the patients:
    “Sometimes we have to get help from police forces to restrain  aggressive patients.” (P. 6).
    “However, the patients themselves help us; those who are conscious help us. Here, we have a lot of patients who are alert and would assist in the patient’s fixation” (P. 4).
    “Ordinary of restraint facilities” is of the other ways related to non-standard control of patients:
    “We do not have special bonds or belts or safety devices that are appropriate for restraining the patients; currently, we use ordinary bands or the bed sheet for this purpose” (P. 4).
    “Staffing limitation for restraint” is of nursing problems in restraining the patients and, according to the participants, it is the main cause of non-standard performance of the procedure:
    “Due to staff insufficiency, if the patient is aggressive or irritable and needs to be fixed, we also ask for the help of other patients and the workers because three or four and, sometimes, five people are needed for doing this, but we are only two nurses in a shift at most” (P. 4).
    “Violent restraint” is of other non-standard approaches of the nurses to restrain the patients. Sometimes, this approach is used in the patients who show much higher resistance to restraint, and includes the use of force and pressure as well as more physical challenge:
    “Sometimes, you’re forced to physically act for patient restraint. I would not say beat, but inevitably you have to sometimes act with physical violence in order to stop the patient” (Part 5).
  - Post-restraint
    The process of physical restraint continues after restraining of the patient, and includes the following three general categories of efforts:
    “Liberating interaction”, which involves stopping or continuing the physical restraint of the patient:
    “After the restraint, we order the patient to lie down; we’re going to evaluate you. If we see that you are able to control your manners, for example, during the dinner or for about half an hour, then we’ll open you” (P. 6).
    “Patient enlightenment”, which consists of explaining about the reasons of physical restraint to the patient:
    “After the restraint, we explain the following to the patient: It is for you, and now whatever you say I will not open you” (P. 11).
    “Assessment of restrained patient”, as one of the most important steps after the restraint, includes the overall assessment of the patient in terms of damage and also assessing the patient to decide on stopping or continuing the restraint:
     “…Next, I go to see what the status of the [restrained] patient is; talking with him/her, I ask him/her about what happened that he/she has been restrained. The patient explains what has happened, then I check whether he/she still needs to be fixed in this shift” (P.6).
    In addition, according to the participants, the duration of holding a patient under restraint is different and dependent on the patient’s condition. But most of the time, it takes around two hours:
    “We usually fix the patients for one to two hours, but it is possible that we fix a patient for a quarter of an hour; it differs from one patient to another”(P. 12). 
    And another participant describes the duration of the patient restraint and its process as follows: 
    “Physical restrain has no special duration and time; if the patient becomes irritable, or lose his/her normal mood, I fix him/her. According to the texts, the maximum period for holding a patient under restraint is two hours. If I see that the patient has become less aggressive, I isolate him/her for about four hours maximum. If I see that he/she is not calm, then I fix him/her this time and inject a sedative drug until he/she becomes calm” (P. 10).
    And finally, this procedure is reported to the doctor:  
     “We fix the patient, write in his/her records and report to the doctor about the event” (Part 10).  
3. *Restraint as a Challenging Subject*
  This theme includes the problems and challenges that the nurses deal with in the physical restraint of patients. It consists of two sub-categories:
  - Opening the restraint
    One of the main challenges that the participants had often talked about was “opening the restraint”. This is mainly due to unfavorable conditions of the ward for physical restraint, and the nurses are forced to restrain the patient in the room in front of other patients and also using primitive tools for physical restraint, which increases the possibility of opening it by the patient or his/her other fellow patients:
     “For example, since we have no seclusion room, our patients are hospitalized in six-bed rooms; six beds in a room, you want to restrain a critical patient in the middle of the six beds, well the other patients would open him/her, because our restraint isn’t safe” (P.2).
  - Resistance to restraint
    Of the other challenges associated with restraint is lack of patient’s acceptance and his/her resistance to the restraint:
    “The patient does not accept that his/her behavior was wrong and that he/she should be closed right now. For example, he/she begins bullying as: I have a superior power! I’ll beat you all! I kill you! and alike” (P. 6).
4. *The restraint Effects on the Spectrum        *
  This is one of the main extracted themes that includes complications and effects of the physical restraint and is comprised of two sub-categories:
  - Negative effects
    One of the consequences of restraint is its negative effects that can be placed in three general sub-categories:
    Damage to the relationship, which given the importance of communication in the care of mentally ill person, has a very serious impact on the patient’s caring process:
     “When you restrain a patient, well the patient blames the nurse and becomes skeptical towards him/her” (P. 2).
    Damage to the patient:
    “It sometimes happens that the patient has been fixed, you open him/her in the morning and see that his/her wrist is sore; this itself creates a problem for the patient” (P. 11).
    Of the other negative consequences of physical restraint, as mentioned by the nurses, is “being affected by the consequences of restraint” consisting of both physical and psychological effects:
     “Because of restraint, the patient ambushes to beat us. Once one of the patients bit my arm and I was hospitalized for 15 days in the hospital; he displaced the muscle totally” (P. 10).
    In addition, one of the participants believes that this procedure can also be a conflicting situation that causes emotional engagement and psychological effects on the nurses:
    “Well, it (restraining a patient) certainly would not have a good feeling; nobody likes to restrain another person. Anyway, he/she is a human being, restraining someone else or any living creature does not have a good feeling; definitely it doesn’t create a good effect” (P. 11).
  - Positive effects of caring
    According to the participants, physical restraint is an accepted intervention in the psychiatric wards and has positive effects such as better patient cooperation as well:
    “When the patient is fixed, he/she gets somewhat calmer, then the relationship that we have already established will be better” (P. 12).
    One of the participants says:
    “Tell him/her: If you take your drugs and eat your meals regularly, then you will not be fixed to the bed, otherwise, we will do it immediately. Patient learns and then he/she will be forced to take his/her drugs to respond to the treatment” (P. 10).

## Discussion

Nurses in the psychiatric wards provide care for the patients in an environment with a potential of stress and a lot of violent and non-adaptive behaviors. To cope with such stresses and behaviors, they use a variety of therapeutic and even non-therapeutic approaches. One of these approaches is physical restraint. In this study, the experiences of 14 psychiatric nurses of psychiatric hospitals in Ahwaz, Iran  were studied.

The first theme is “restraint as a multi-purpose procedure. It appears that physical restraint is of the main nursing interventions to manage and control the patients in psychiatric wards.  It is used with different purposes, including the positive attitude of the nurses to this procedure. Nurses’ attitude to physical restraint is considered as one of the main reasons for the various uses of this approach.^18^ Among the applications of physical restraint is to use it as a means to control high-risk and aggressive patients. Generally, management of non-adaptive behavior, according to the participants, is one of the main applications of this procedure. Application of physical restraint as a means to control non-adaptive behavior of patients has been emphasized in several studies. These studies report that coercive tools are mainly used as a means of control in situations where a patient’s violent behavior threatens the safety of others.^19^

Moreover, “treatment auxiliary” is of other various applications of restraint, in which physical restraint is used as an auxiliary device until the onset of the therapeutic effects of sedative drugs in some patients. In addition, according to a participant, in cases such as obsessive-compulsive symptoms in the patients, the nurse uses restraint as a way to cope with these symptoms; this helps the patients to control their obsessive behavior. Coercive interventions are used not only to help, treat or cure but also to control the psychotic patients.^19^ Physical restraint can also be used for helping psychiatric patients who are unable to control their emotions and behaviors.^20^

The second theme, “processing of restraint”, is of the nurses’ physical restraint approaches, which includes three general stages: actions before, during and after the restraint.

“Before restraint” action includes assessment of the patient to restrain or not. It mainly consists of determining the necessity of physical restraint of a patient. Therefore, the nurse, using his/her experience and intuition, and also according to the diagnosis of the patient’s disorder, defines a patient’s need for restraint. Learning to identify when physical restraint is the only safe method is an important skill for the nurses in mental health care institutions.^21^ The diagnosis associated with rapid restraining of the patient in this study was antisocial personality disorder. Other researchers reported that most of the restrained patients were diagnosed as having schizophrenia, personality disorder, acute psychosis, mania, and substance abuse.^22^

“Preparing the patient” that includes injecting of tranquilizers to reduce resistance in the patient, is one of the other actions before the restraint. Injection of psychotropic drugs is another options for dealing with an emergency situation to calm down the patients during aggression or provocation.^23^ In the study of Reghabi, restrained adults and adolescents received a PRN medication before the restraint.

“Action during the restraint”, as another stage of restraint, in highly important in the proper implementation of the process. Approaches of nurses in this stage include surrounding the patient, reducing his/her resistance, and safety restraint of the patient in order to minimize the possible damage to both the patients and the staff. For this purpose, nurses apply methods such as using more persons to restrain the patient, distracting the patient’s thoughts, and attempting to restrict the movements of his/her limbs simultaneously while considering the safety of restraint and reducing harm to the patient. During the physical restraint, two main goals are to be met for successful intervention: First, the physical security of the patient, other patients and care providers must be preserved. The second goal is that the patient’s dignity, humanity and comfort throughout the procedure of restraint must be advocated actively.^21^ Nevertheless, in attempting to reduce resistance and safety restraint of the patient, the nurses in the present study used non-standard approaches, including the use of non-nursing staff, violent restraint of the patient, and also using ordinary facilities and equipment. These approaches to physical restraint were fundamentally affected by nursing shortage in the ward. It is argued that using physical restraint, mainly due to improper applying of techniques and inadequate understanding of the needs of patients, may not be affective in ensuring the patient’s safety and comfort.^3^ Therefore, all staff in acute psychiatric care wards require excessive knowledge about the policies and procedures related to the use of restraint.^21^ In a study in Iran, there were significant differences between the performance of nurses in restraining the patient and the existing standards.

The process of physical restraint continues even after the restraint of the patient. It includes nursing care, interaction to assess the patient to ensure prevention of damage to him/her, interaction with the patient for the commitment and release of restraint and also explaining to the patient about the reasons and the need to restrain him/her. Chien and colleagues (2005) believe that a licensed employee should assess the need for restraint in an hour of practice, and the patient should be continuously monitored by the personnel during the restraint and evaluated to remove the restraint as soon as possible.

The third theme is “restraint as challenging subject”. Restraint as a physical intervention has aggressive and unpleasant nature and, obviously, its application in patients is often associated with some challenges. “Removing of restraint” is one of these challenges discussed by the participants. This occurs mainly due to restraining the patient to the bed and next to other patients, and also applying non-standard tools such as using band for restraint. While, according to the researchers, use of appropriate and standard tools is necessary for physical restraint of the patients.^21^

Of other challenges associated with the restraint is “the patient’s opposition or resistance” against it, which occurs because of the restrictive nature of restraint and the patient’s perception of being punished. Restraint process is considered as intensive physical intervention for patients.^21^ Studies have shown that many patients have reported mechanical restraint and seclusion  as a negative intervention, a punishment and even a form of retribution.^12^ These views of patients can underlay their opposition and resistance to restraint.

The last theme derived is the “the restraint effects on the spectrum” that includes negative and positive effects. Negative effects include damage to the relationship, damage to patients and affected nurse. Several studies have shown adverse effects of restraint. In fact, one of the reasons of opposition to this procedure is unpredictable incidence of damage and complications in both the patient and nurse.^10^ Restraint increases the risk of physical damage and even death in patients and is of the most common causes of personnel damage.^6^

In addition, this situation, according to some participants, is a dilemma that affects nurses emotionally and mentally. Marangos-Frost and Wells reported physical restraint as a decision dilemma.^13^

One important issue among the consequences of restraint is the potential damage of this procedure on the nurse-patient relationship. Often psychiatric care is dependent on an alliance between the patients and the staff, and use of restraint or seclusion  can affect this therapeutic alliance.^24^

Another outcome associated with restraint is the “positive effects of care”. These effects are incentives for continued use of restraint, and despite the complications and ethical challenges associated with restraint, they are also the causes of staff’s positive attitude toward this approach. There are few studies  showing the positive effects of physical restraint.^25^ It is believed that physical restraint is a method to  control people externally in order to make them  learn. To some extent, it has internal control over their socially acceptable behavior.^26^ However, the idea that inhibition can never be a therapeutic tool needs more evaluation.^21^

The limitation of this study includes the small sample size, which ended with limited applicability of the results.

## Conclusion

The nurses participating in this study expressed physical restraint as one of the main strategies to control and manage psychiatric patients. Although this method is not to the satisfaction of neither patients nor nurses, nurses are inevitably forced to use it. However, in order to apply it, respectful and appropriate communication with the patients is essential. Also, since physical restraint is known as a challenging intervention throughout the world, training the nurses to find alternative ways, resulting in the reduction of physical restraint of patients, is recommended.
